# Development of a prognostic risk score to aid antibiotic decision-making for children aged 2-59 months with World Health Organization fast breathing pneumonia in Malawi: An Innovative Treatments in Pneumonia (ITIP) secondary analysis

**DOI:** 10.1371/journal.pone.0214583

**Published:** 2019-06-20

**Authors:** Eric D. McCollum, Siobhan P. Brown, Evangelyn Nkwopara, Tisungane Mvalo, Susanne May, Amy Sarah Ginsburg

**Affiliations:** 1 Eudowood Division of Pediatric Respiratory Sciences, Department of Pediatrics, School of Medicine, Johns Hopkins University, Baltimore, Maryland, United States of America; 2 Department of International Health, Bloomberg School of Public Health, Johns Hopkins University, Baltimore, Maryland, United States of America; 3 Department of Biostatistics, University of Washington Clinical Trial Center, Seattle, Washington, United States of America; 4 Save the Children, Fairfield, Connecticut, United States of America; 5 University of North Carolina Project, Lilongwe Medical Relief Fund Trust, Lilongwe, Malawi; 6 Department of Pediatrics, School of Medicine, University of North Carolina at Chapel Hill, Chapel Hill, NC, United States of America; Canberra Hospital, AUSTRALIA

## Abstract

**Background:**

Due to increasing antimicrobial resistance in low-resource settings, strategies to rationalize antibiotic treatment of children unlikely to have a bacterial infection are needed. This study’s objective was to utilize a database of placebo treated Malawian children with World Health Organization (WHO) fast breathing pneumonia to develop a prognostic risk score that could aid antibiotic decision making.

**Methods:**

We conducted a secondary analysis of children randomized to the placebo group of the Innovative Treatments in Pneumonia (ITIP) fast breathing randomized, controlled, noninferiority trial. Participants were low-risk HIV-uninfected children 2–59 months old with WHO fast breathing pneumonia in Lilongwe, Malawi. Study endpoints were treatment failure, defined as either disease progression at any time on or before Day 4 of treatment or disease persistence on Day 4, or relapse, considered as the recurrence of pneumonia or severe disease among previously cured children between Days 5 and 14. We utilized multivariable linear regression and stepwise model selection to develop a model to predict the probability of treatment failure or relapse.

**Results:**

Treatment failure or relapse occurred in 11.5% (61/526) of children included in this analysis. The final model incorporated the following predictors: heart rate terms, mid-upper arm circumference, malaria status, water source, family income, and whether or not a sibling or other child in the household received childcare outside the home. The model’s area under the receiver operating characteristic score was 0.712 (95% confidence interval 0.66, 0.78) and it explained 6.1% of the variability in predicting treatment failure or relapse (R^2^, 0.061). For the model to categorize all children with treatment failure or relapse correctly, 77% of children without treatment failure or relapse would require antibiotics.

**Conclusion:**

The model had inadequate discrimination to be appropriate for clinical application. Different strategies will likely be required for models to perform accurately among similar pediatric populations.

## Introduction

According to the most recent global estimates, the greatest numbers of children under five years old who die each year live in sub-Saharan Africa [1]. Presumed bacterial pneumonia is the leading cause of child mortality with 490,000 annual deaths in Africa, and 921,000 annual deaths globally [1]. World Health Organization (WHO) guidelines define child pneumonia as a clinical syndrome with either fast breathing, lower chest wall indrawing, or general clinical danger signs in children aged 2–59 months with cough or difficult breathing [2]. Introduction of these pragmatic guidelines have markedly improved antibiotic access, contributing substantially to the decline in child pneumonia mortality over the past two decades in low- and middle-income countries (LMICs) [3].

The more recent introduction of vaccines for *Haemophilus influenzae* and *Streptococcus pneumoniae*, two important etiologies for fatal bacterial pneumonia, to the sub-Saharan African region, have additionally accelerated this mortality decline [4]. However, these vaccines are also speculated to be driving the transition of pneumonia etiology away from more severe bacterial and viral co-infections toward less severe viral causes [5, 6]. This epidemiologic shift is suggested to be reducing the specificity of the WHO guidelines such that increasing numbers of children with non-bacterial pneumonia are unnecessarily treated with antibiotics [7]. In parallel to this is the concerning rise of antimicrobial resistance (AMR) in LMICs [8]. Recent estimates indicate that high proportions of pathogens in sub-Saharan Africa have developed AMR to first-line antibiotics [9–12]. Innovative, pragmatic strategies that improve antibiotic stewardship among children at low risk of mortality with presumed pneumonia and are feasible to implement in LMICs are a priority [13].

In this secondary analysis of the Innovative Treatments in Pneumonia (ITIP) fast breathing randomized, controlled, noninferiority trial [14], we sought to develop a novel prognostic risk score to aid antibiotic decision making by leveraging a unique dataset that included blinded placebo treatment of children aged 2–59 months with WHO fast breathing pneumonia in the sub-Saharan African country of Malawi. The objective of this score would be to identify those children at initial clinical presentation who are at low risk of treatment failure or relapse without antibiotic treatment. This score would serve to complement current WHO guidelines in deciding whether or not to prescribe antibiotics to low risk children in LMICs with pneumonia characterized by fast breathing only and who are without other established risk factors associated with poor outcomes.

## Materials and methods

### Setting

ITIP (ClinicalTrials.gov registration: NCT02760420) was a prospective, double-blind, randomized, controlled, noninferiority trial of 1,126 HIV-uninfected children aged 2–59 months with WHO fast breathing pneumonia conducted between 2016–2017 in Lilongwe, Malawi. The trial’s primary aim was to determine if placebo treatment was noninferior to three days of amoxicillin treatment. This secondary analysis utilized data from children enrolled in the placebo group of this trial with outcome data. ITIP1 took place in the capital city of Lilongwe within the outpatient department of Bwaila District Hospital (BDH), which provides primary healthcare, and the outpatient department and pediatric wards of Kamuzu Central Hospital (KCH), the tertiary referral hospital for the central region of Malawi.

### Participants

All caregivers of participant children provided written informed consent. The ITIP1 fast breathing pneumonia case definition was consistent with WHO guidelines, and as shown in the Table 1, eligibility criteria included low risk children aged 2–59 months with cough or difficult breathing <14 days who also had fast breathing for age. Enrolled children who did not meet all eligibility criteria were excluded from this secondary analysis, as were children missing the outcome or key variables.

**Table 1:** Study eligibility criteria.

Eligibility criteria	
Inclusion criteria	• 2–59 months of age • Cough <14 days or difficulty breathing Fast-breathing for age, defined as a respiratory rate ≥50 breaths per minute for children 2 to <12 months old or ≥40 breaths per minute for children ≥12 months old
Exclusion criteria	• Severe respiratory distress (grunting, nasal flaring, head nodding, and/or chest-indrawing) • Hypoxemia, an arterial oxyhemoglobin saturation <90% in room air, as assessed non-invasively by a pulse oximeter • Resolution of fast breathing after bronchodilator challenge, if wheezing at screening examination • General danger signs (lethargy or unconsciousness, convulsions, vomiting everything, inability to drink or breastfeed, stridor when calm) • HIV-1 seropositivity • HIV-1 exposure (a child <24 months of age with a HIV-infected mother) • Severe acute malnutrition (weight for height/length < -3 SD, mid-upper arm circumference <11.5 cm, or peripheral edema) Possible tuberculosis (coughing ≥14 days) • Anemia with hemoglobin <8.0 g/dL • Severe malaria (positive malaria rapid diagnostic test with any general danger sign, stiff neck, abnormal bleeding, clinical jaundice, or hemoglobinuria) • Known allergy to penicillin or amoxicillin • Receipt of an antibiotic treatment in the 48 hours prior to the study • Hospitalized within 14 days prior to the study • Living outside the study area • Any medical or psychosocial condition or circumstance that, in the opinion of the investigators, would interfere with the conduct of the study or for which study participation might jeopardize the child’s health • Any non-pneumonia acute medical illness which requires antibiotic treatment per local standard of care • Participation in a clinical study of another investigational product within 12 weeks prior to randomization or planning to begin participation during this study • Prior participation in the study during a previous pneumonia diagnosis

### Data collection

ITIP study clinicians, called clinical and medical officers, as well as nurses trained to a standardized screening protocol under the supervision of an ITIP study pediatrician, were based at BDH and KCH, and assessed children aged 2–59 months with fast breathing pneumonia for trial eligibility. If children were found eligible, they were randomized double-blinded in a 1:1 ratio to three days of either placebo or amoxicillin treatment. To optimize child safety, all study children were monitored at KCH for 2–8 hours after enrollment. Study staff discontinued monitoring after two hours for children without fever who were breathing slower than at enrollment. Those children <6 months old, with a mid-upper arm circumference (MUAC) 11.5–13.5cm, or febrile with a negative malaria test were monitored overnight. At any point during monitoring, if a child clinically deteriorated, then parenteral antibiotics were initiated, and the child was classified as treatment failure (Table 2).

**Table 2:** Treatment failure and relapse criteria.

Treatment failure criteria	
Anytime on or before Day 4	• Severe respiratory distress (grunting, nasal flaring, head nodding, and/or chest-indrawing) • Hypoxemia (arterial oxyhemoglobin saturation <90% in room air, as assessed non-invasively by a pulse oximeter) • General danger sign (lethargy or unconsciousness, convulsions, vomiting everything, inability to drink or breastfeed, stridor while calm) Missing ≥2 study drug doses due to vomiting • Change in antibiotics prescribed by a study clinician • Hospitalization due to pneumonia (if not initially admitted) • Prolonged hospitalization or re-admission due to pneumonia (if initially admitted) • Death
On Day 4 only	• Axillary temperature ≥38°C in the absence of a diagnosed co-infection with fever symptoms (e.g., malaria)
Relapse criteria	
Anytime after Day 4	• Recurrence of signs of pneumonia • Signs of severe disease

Study staff contacted caregivers of children not under hospital monitoring on Days 1–3 by phone, and assessed children in person at the study clinic or at home on Days 2–4 and Day 14. At all in person assessments, whether scheduled or not, study staff evaluated children for treatment failure or relapse, and study drug adherence.

### Outcome definitions

Treatment failure was defined as occurring anytime on or before study Day 4. For children without treatment failure on Day 4, they were considered to have relapsed if they were found to have recurrence of signs of pneumonia or severe disease on or before Day 14 (Table 2).

### Analysis

Continuous data were summarized with means and standard deviations, and differences between children with and without treatment failure or relapse were tested with a student’s t-test. For categorical data, we gave the number and proportion falling into each category and used Pearson chi-squared tests to compare proportions.

We developed a prognostic model for the probability of treatment failure or relapse using multivariable linear regression through stepwise model selection [15]. Linear regression was used to allow direct estimate of the failure probability, with robust standard error to accommodate the binary nature of the outcome, reasonable with the given sample size. The same model was used in the primary analysis of the study. Variables with a significance level of 0.10 or lower entered the model; those with a significance level below 0.20 remained in the final model. We chose cut-points on the high end of what is used in stepwise model building as we hoped to maximize the predictive performance of the model rather than focusing on the interpretation of the selected variables [16, 17]. Covariates to be considered for the prognostic model were selected *a priori* based on previous knowledge supported either by published literature or expert input. For continuous variables, the functional form included in the model building was based on bivariate modeling of each factor with the outcome. For each, we considered a linear form, a spline, and categorizations selected *a priori*; when the spline suggested a polynomial fit, one was considered as well. The form with the highest Akaike Information Criterion (AIC) was used in the model building [18]. The use of non-linear relationships such as splines and quadratics prioritized the development of the best fitting model regardless of interpretation complexity, under the assumption that the model could be implemented during care using electronic decision support aids. Missing covariates were minimal, with the exception of family income, which was explicitly modeled. We used R^2^, AIC, and area under the receiver operating characteristic (AUROC) to evaluate the model performance. We also assessed the model under the presumption that the prognostic risk score would only be acceptable for implementation by policy makers, healthcare providers, and caregivers if all children with treatment failure or relapse were correctly identified for antibiotic treatment. Therefore, we additionally evaluated the model’s performance by determining what percentage of children the model would falsely predict with likely treatment failure or relapse, and therefore receive antibiotic treatment, in order for the model to identify all children who would become a true treatment failure or relapse case without antibiotics. SAS 9.4 was used for data management and the creation of calculated variables, while baseline data summaries were produced in R 2.14.1. Predictive model building was done in STATA 14.2.

### Ethics

The Western Institutional Review Board in Washington, USA, the College of Medicine Research and Ethics Committee in Blantyre, Malawi, and the Malawi Pharmacy, Medicines and Poisons Board approved this study.

## Results

**Fig 1:**
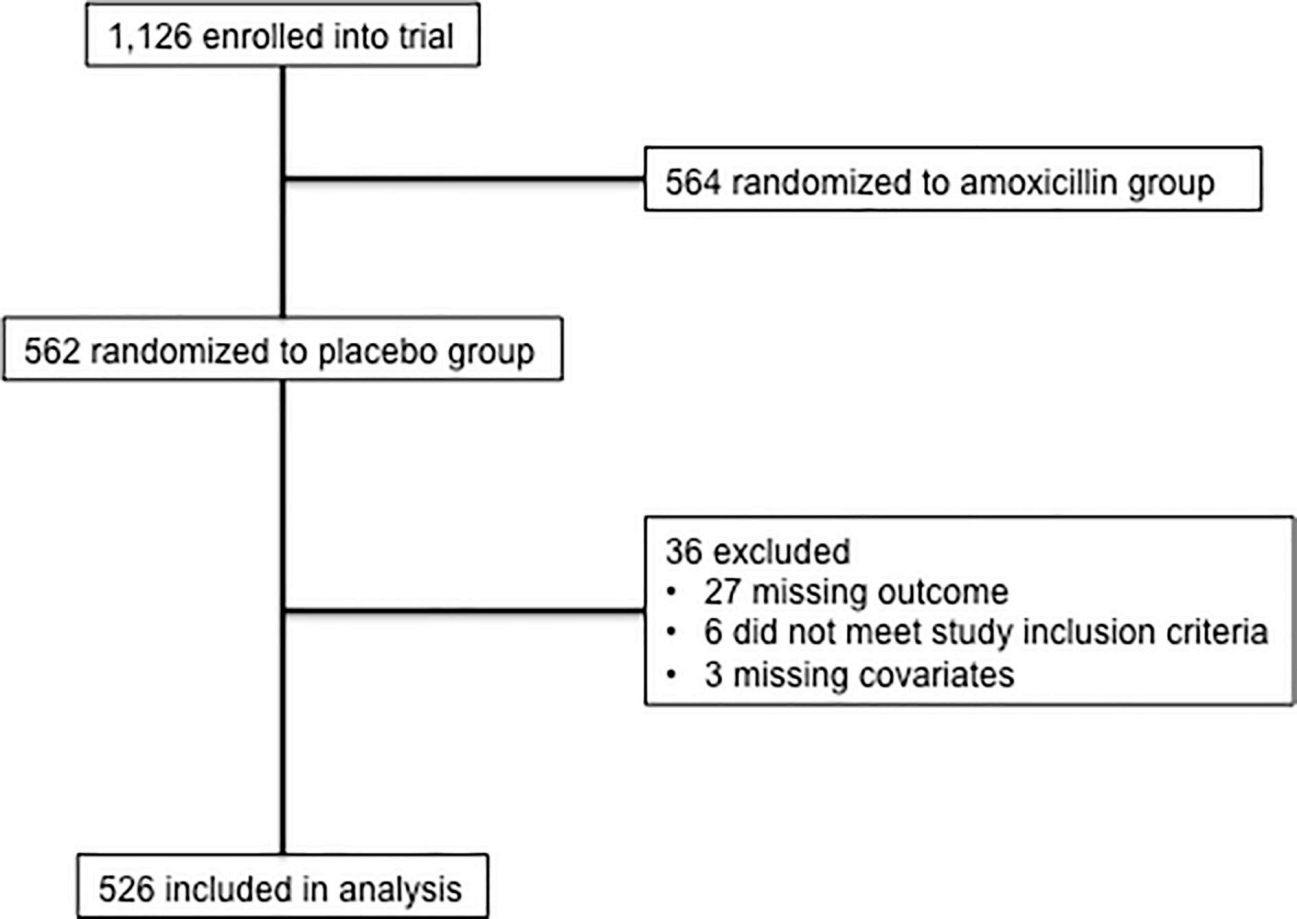
Study schema.

In ITIP, 562 children with fast breathing were randomized to the placebo group. Of these, 27 were missing treatment outcome and were excluded from this secondary analysis. A further six were excluded because they did not meet inclusion/exclusion criteria, three others were dropped because of missing covariates (Fig 1, Table 3). Overall, 11.5% (61/526) of children treated with placebo met treatment failure or relapse criteria in this dataset. Placebo recipients with treatment failure or relapse, compared to those without treatment failure or relapse, were 5.0 months younger (17.1 vs 22.1 months, p-value = 0.008), their MUAC was 0.4 cm smaller on average (14.7 cm vs 15.1 cm, p-value = 0.009), and 11.3% more were malaria negative according to rapid antigen testing (98.4% vs 87.1%, p-value = 0.018, Table 3). In addition, there was weak evidence towards a higher proportion of children with a peripheral arterial oxyhemoglobin saturation 90–95% (6.6% vs 1.9%, p-value = 0.081), a higher average respiratory rate (51.7 vs 49.8 breaths/minute, p-value = 0.061), and a difference in categories of water source between children with and without treatment failure or relapse (p-value = 0.083).

**Table 3:** Baseline characteristics of placebo recipients according to outcome.

	No Treatment Failure or Relapse	Treatment Failure or Relapse	
	N = 465	N = 61	p-value
Age in months, mean (SD)	22.1 (15.0)	17.1 (13.3)	0.008
Female, n (%)	255 (54.8%)	33 (54.1%)	0.978
PCV doses, n (%)			
0	172 (37.0%)	19 (31.1%)	0.264
1	6 (1.3%)	0 (0.0%)	
2	22 (4.7%)	6 (9.8%)	
3	265 (57.0%)	36 (59.0%)	
Pentavalent vaccine doses, n (%)			
0	171 (36.8%)	19 (31.1%)	0.351
1	7 (1.5%)	0 (0.0%)	
2	25 (5.4%)	6 (9.8%)	
3	262 (56.3%)	36 (59.0%)	
Mother's education, n (%)			
None/Primary/Missing	214 (46.0%)	25 (41.0%)	0.544
Secondary/Tertiary	251 (54.0%)	36 (59.0%)	
Maternal HIV status, n (%)			
Negative	435 (93.5%)	57 (93.4%)	0.780
Positive	17 (3.7%)	3 (4.9%)	
Unknown	13 (2.8%)	1 (1.6%)	
Mother's age in years, mean (SD)	25.6 (5.2)	25.7 (5.2)	0.923
Family income in thousands of Malawi kwacha, mean (SD)	59.8 (74.5)	87.9 (94.6)	0.118
Log family income in Malawi kwacha, mean (SD)	10.6 (0.8)	11.0 (0.8)	0.016
Missing family income, n (%)	271 (58.3%)	29 (47.5%)	0.146
Weight-for-height Z-score, mean (SD)	0.4 (1.2)	0.6 (1.3)	0.373
MUAC in cm, mean (SD)	15.1 (1.2)	14.7 (1.1)	0.009
Oxygen saturation, mean (SD)	98.7 (1.4)	98.6 (1.6)	0.681
Oxygen saturation ≤95%, n (%)	9 (1.9%)	4 (6.6%)	0.081
Dehydration, n (%)	9 (1.9%)	3 (4.9%)	0.312
Malaria rapid diagnostic test positive, n (%)	60 (12.9%)	1 (1.6%)	0.018
Respiratory rate in breaths per minute, mean (SD)	49.8 (7.3)	51.7 (7.2)	0.061
Respiratory rate category^1^, n (%)			
Fast	324 (69.7%)	41 (67.2%)	0.868
Very fast	131 (28.2%)	19 (31.1%)	
Extremely fast	10 (2.2%)	1 (1.6%)	
Temperature in°C, mean (SD)	37.4 (1.0)	37.3 (1.0)	0.410
Diarrhea, n (%)	42 (9.0%)	7 (11.5%)	0.702
Heart rate in beats/min, mean (SD)	141.8 (17.8)	143.3 (13.6)	0.459
Hemoglobin in g/dL, mean (SD)	10.9 (1.2)	10.9 (1.3)	0.858
Lives with smoker, n (%)	45 (9.3%)	8 (12.7%)	0.540
Water source, n (%)			0.083
Other	17 (3.7%)	2 (3.3%)	
Piped	413 (88.8%)	59 (96.7%)	
Well	35 (7.5%)	0 (0.0%)	
Cooking smoke inside, n (%)	440 (94.6%)	58 (95.1%)	0.878
Sibling receives care out of home, n (%)	239 (51.4%)	38 (62.3%)	0.143

SD indicates standard deviation; PCV, pneumococcal conjugate vaccine; HIV, human immunodeficiency virus.

^1^For ages 2–11 months: 50–59, 60–69, and ≥70 breaths/minute. For 12–59 months: 40–49, 50–59, and ≥60 breaths/minute.

In Table 4 we report a model that predicts the probability of treatment failure or relapse among children receiving placebo. Heart rate terms, MUAC, malaria status, water source, family income, and whether or not a sibling or other child in the household received childcare outside the home were all included in the final predictive model. Specifically, an increase in MUAC, testing malaria positive, the categories of other water source and well water, relative to the piped water referent category, and missing family income were all associated with a lower risk of treatment failure or relapse. On the other hand, increasing family income and having a sibling receive childcare outside the home were associated with a statistically significant higher risk of treatment failure or relapse. Failure rates were the highest for heart rates between 120 and 170, peaking near 145 beats per minute, and reduced for higher or lower baseline heart rates. The model equation is:

$$\begin{array}{l} P_{failure\;or\;relapse}\\ \;=-1.691-0.010I_{well\;water}-0.14I_{other\;water}-0.003x_{MUAC}\\ \;+0.227x_{heartrate/10}-0.008{x^{2}}_{heartrate/10}-0.082x_{malaria}\\ \;+0.062x_{in(family\;income)}-0.036I_{missing\;income}+0.045I_{sibling\;outside\;childcare} \end{array}$$

**Table 4:** Model predicting the risk of treatment failure or relapse among placebo recipients.

Effect	Estimated Risk Difference	95% CI	P value
Piped water	Reference		
Other water source	-0.014	-0.152, 0.125	0.846
Well water	-0.100	-0.135, -0.065	<0.001
MUAC	-0.003	-0.005, -0.001	0.008
Heart rate (10 beats per minute)	0.227	0.108, 0.346	<0.001
(Heart rate/10)^2^	-0.008	-0.012, -0.004	<0.001
Malaria	-0.082	-0.128, -0.037	<0.001
Log family income	0.062	0.009, 0.116	0.022
Missing family income^1^	-0.036	-0.091, 0.018	0.192
Sibling receives childcare outside home	0.045	-0.008, 0.098	0.099
Intercept	-1.691	-2.738, -0.643	0.002

CI indicates confidence interval; MUAC, mid-upper arm circumference.

^1^Missing compared to median known income.

**Fig 2:**
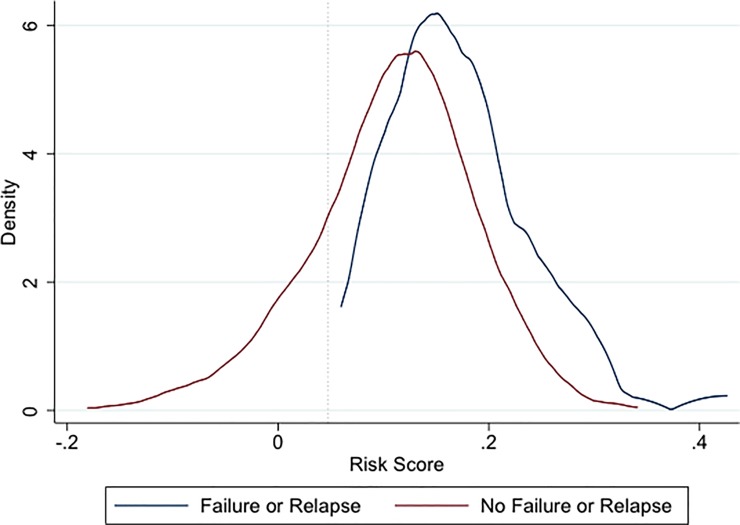
Risk prediction score plot.

We found that this model explained just 6.1% of the variability in predicting treatment failure or relapse (R^2^, 0.061) and achieved a goodness of fit, as measured by the c-statistic (AUROC), of 0.712 (95% confidence interval 0.66, 0.78). Both of these model fit indicators imply modest model performance overall. Furthermore, we found that in order to correctly classify all children who developed treatment failure or relapse in this dataset, we would need to incorrectly treat 77% of those children with antibiotics who did not develop treatment failure or relapse without antibiotic treatment. While we considered applying less restrictive criteria to allow some children to develop treatment failure or relapse without antibiotics, the substantial overlap in prognostic risk scores across the two outcome groups meant–even with less restrictive criteria–the proportion of false positives would change little. A risk prediction score plot visually depicts the density of the relative overlap between children with and without treatment failure or relapse, again suggesting that the model inadequately discriminates between these two outcomes (Fig 2).

## Discussion

We leveraged a unique dataset of placebo treated Malawian children in an effort to develop a novel prognostic risk score to complement current WHO guidelines in deciding which 2–59 month olds with fast breathing do or do not require antibiotics in LMICs. Given incorrect non-treatment of a bacterial infection with antibiotics can be fatal in settings where many children may only access healthcare once, a prognostic risk score to facilitate a “watch and wait” antibiotic treatment approach requires high accuracy for it to be acceptable to caregivers, healthcare providers, or policy makers in LMICs. It is against this backdrop that we found our model to be inadequate for clinical application as the model performed only modestly, and did not have sufficient discrimination between outcomes. Application of our model would result in the incorrect treatment of 77% of fast breathing children with antibiotics in order to ensure no fast breathing child who would otherwise progress to treatment failure or relapse went without treatment. In other words, use of this model would theoretically reduce the number needed to treat to prevent one treatment failure or relapse episode from 33 to 26.

Prognostic risk scores have been implemented in resource rich hospital settings to identify children at risk of death [19], and similarly designed scores are now emerging from LMICs [20]. Scores from hospitalized children have been developed using databases from Columbia, Ghana, India, Kenya, Malawi, Mexico, Pakistan, South Africa, Vietnam, and Zambia [21–25]. Only one score from South Africa has been successfully externally validated [21, 23], and only one score in Malawi has been implemented, with results suggesting a reduction in hospital mortality post-implementation [26]. Notably, the South African RISC score, which included one score for HIV-infected and another score for HIV-uninfected children with pneumonia, achieved a c-statistic of 0.77 (HIV-infected model) and 0.92 (HIV-uninfected model) during development, a c-statistic of 0.74 to 0.92 (HIV-uninfected) and 0.66 to 0.73 (HIV-infected) during internal validation, and a c-statistic of 0.72 during external validation on a Malawi dataset [21, 23]. While our prognostic risk score achieved a similar c-statistic of 0.71, identifying children at low risk for poor outcomes requires much higher discrimination to be safely implementable.

Interestingly, this same standard may not apply to adult pneumonia populations. In a large study of hospitalized adults in the United States a prediction score was developed and validated to stratify patients into categories of low or high risk of death [27]. The authors reported a c-statistic of 0.84 during score development and 0.83 during external validation, and concluded that their score accurately predicted adults with pneumonia who are at low risk of death and did not require hospitalization [27]. Despite achieving high accuracy, seven deaths were still included in the model’s low risk category [27]. Furthermore, to achieve this accuracy, the score was comprised of 20 characteristics including comprehensive medical histories, six invasive blood samples including an arterial blood gas, and chest radiography [27]. This level of diagnostic complexity is not feasible in the vast majority of LMIC primary care settings where detailed medical histories are rarely available and laboratory and radiographic access is limited. Nevertheless, given that innovative approaches to improve antibiotic stewardship in LMICs remain a top priority, prognostic risk scores to identify low risk patients that can be managed without antibiotics warrants continued exploration in similar adult populations or other pediatric pneumonia patient groups.

Children with an acute illness characterized by only fast breathing may be an especially challenging population to develop and validate an accurate prognostic risk score. Fast breathing among children under the age of five years is non-specific with many possible causes, some of which are pathologic and some of which are benign. The spectrum of more pathologic causes includes bacterial lower respiratory tract infection, which requires antibiotics, while the spectrum of more benign causes includes anxiety, which does not require antibiotics. In addition, counting respirations in a child can be a vexing task to even the most skilled clinicians, requiring one full minute of focused attention on the chest’s subtle rise and fall in an often moving, agitated, anxious, or crying child. Many caregivers are reluctant to fully expose the chest of their child and many time-constrained healthcare providers do not consistently count respirations for a full minute, both of which further reduce the reliability of respiratory rate counts [28, 29]. These issues pose substantial barriers to creating a sufficiently discriminative score from demographic variables and clinical markers. One previous study, also from Malawi, sought to develop a prognostic risk score to predict treatment failure among 2–59 month old children with fast breathing [30]. In this study trained community health workers diagnosed children with fast breathing and prescribed antibiotics [30]. While our model performed better in comparison, the previous study’s authors also concluded that their model had an unacceptably low discrimination (c-statistic 0.56) that prevented clinical implementation [30]. Taken together, these results suggest that a predictive score among a population of fast breathing children is unlikely to be sufficiently accurate without collecting invasive biospecimens or imaging.

This secondary analysis has several limitations. First, the ITIP fast breathing trial excluded most children with known risk factors for death such that the dataset may not be representative of all Malawian children with fast breathing. However, current WHO guidelines require hospitalization of high risk children with fast breathing, and this population was not our target group [2]. Second, the outcomes of treatment failure and relapse are themselves relatively non-specific for death, and many children meeting criteria for these endpoints improve without any intervention [31]. Given no placebo recipients participating in this trial died, these endpoints were the only outcomes available. Additionally, treatment failure and relapse remain clinically relevant in real-world practice as current WHO guidelines recommend treatment changes when they occur [2]. Third, treatment failure and relapse occurred relatively infrequently, and this limited our ability to evaluate all potential risk factors in our model. Additional studies from Pakistan that include placebo treatment of fast breathing children have been completed, and future data pooling for a meta-analysis may help to address this limitation [32, 33]. Lastly, since the primary aim of the project was to develop a predictive model and assess its accuracy, we did not adjust for multiple comparisons or use any other methods to reduce the risk of type I error. Indeed, the model building methods we utilized are known to result in biased point estimates and inflated p-values, so any direct interpretation of those metrics should be done with caution [34].

In conclusion, while the outcomes of this study’s placebo group shed light on the natural history of children with WHO fast breathing pneumonia in a sub-Saharan African setting, model development to accurately predict treatment failure or relapse was unsuccessful. Next steps may include data pooling with other similarly unique datasets or development of prognostic risk scores based on minimally invasive biospecimens or imaging that could be feasible for implementation in LMICs.

## Supporting information

S1 File Data. (CSV)

S2 File Data discionary. (XLSX)
